# Effectiveness of the Bilateral and Bilevel Erector Spinae Plane Block (ESPB) in Pediatric Idiopathic Scoliosis Surgery: A Randomized, Double-Blinded, Controlled Trial

**DOI:** 10.1097/BPO.0000000000002707

**Published:** 2024-05-01

**Authors:** Małgorzata Domagalska, Bahadir Ciftsi, Piotr Janusz, Tomasz Reysner, Przemysław Daroszewski, Grzegorz Kowalski, Katarzyna Wieczorowska-Tobis, Tomasz Kotwicki

**Affiliations:** * Chair and Department of Palliative Medicine, Poznan University of Medical Sciences; Departments of ‡ Spine Disorders and Pediatric Orthopedics; § Organization and Management in Health Care, Poznan University of Medical Sciences, Poznań, Poland; † Department of Anesthesiology and Reanimation, Istanbul Medipol University, Istanbul, Turkey

**Keywords:** ESPB, erector spinae plane block, MEP, motor‐evoked potentials, nerve block, scoliosi

## Abstract

**Background:**

This study aimed to compare the effect of the ultrasound-guided bilateral and bilevel erector spinae plane block (ESPB) on pain scores, opioid requirement, intraoperative motor-evoked potentials (MEPs), and stress response to surgery expressed by the neutrophil-to-lymphocyte ratio (NLR) and platelet-to-lymphocyte ratio (PLR) versus standard analgesia methods following idiopathic scoliosis surgery.

**Methods:**

This was a prospective, double-blinded, randomized controlled trial. Sixty patients aged 10 to 18 years and physical status ASA 1 or 2 were randomized into 2 equal groups, each receiving an ESPB or sham block. The primary outcome was the pain scores (Numerical Rating Scale, NRS) within 48 hours after spinal correction and fusion surgery for idiopathic thoracic scoliosis. The secondary outcomes were total opioid consumption, NLR, and PLR levels at 12 and 24 hours postoperatively and intraoperative MEPs.

**Results:**

ESPB patients presented lower NRS scores, signifying less pain, at all time points (30, 60, 90, 120 min; and 6, 12, 24, and 48 h after surgery), all *P*<0.0001. The total opioid consumption, the incidence of nausea or vomiting, and the need for remifentanil and propofol during surgery were significantly lower in the ESPB group. The surgery-induced stress response expressed by NLR and PLR was considerably lower in the ESPB group. ESPB did not affect the intraoperative MEP’s amplitude.

**Conclusions:**

ESPB is effective for postoperative analgesia, can reduce opioid consumption in patients undergoing scoliosis surgery, and reduces the stress response to surgery. ESPB does not interfere with neuromonitoring.

**Level of Evidence:**

Level I.

Posterior spinal correction and fusion surgery can cause severe postoperative pain that requires opioids for perioperative analgesia. Acute postoperative pain is thought to aggravate postoperative inflammatory response,^1,2^ which risks provoking a systemic inflammatory response syndrome (SIRS). SIRS was reported to complicate patients undergoing scoliosis surgery.^3^ Poorly controlled SIRS can lead to dysregulation of anti-inflammatory and proinflammatory cascades, resulting in severe immunosuppression and predisposing to infections. This can cause sepsis, septic shock, and multiorgan dysfunction syndrome (MODS).^4^

Scoliosis surgery presents an inherent risk of damage to neural structures and may result in postoperative neurological deficits. Intraoperative neuromonitoring techniques, especially transcranial motor-evoked potentials (MEPs), have been developed to improve the safety of scoliosis surgical procedures.^5^ The anesthesia techniques should be optimized to reduce the impact on MEPs during spine surgery.

Therefore, safe and effective perioperative analgesic methods that reduce the stress response to surgery and do not interfere with neuromonitoring are essential for an uneventful course and early recovery.

Regional anesthesia has gained popularity in scoliosis surgery. Intrathecal morphine was often added to general anesthesia to prevent pain after major spine surgeries.^6^ However, intrathecal morphine is often associated with respiratory depression, pruritus, nausea, and vomiting.^7^ These side effects limit the routine use of intrathecal morphine for pain management in spine surgery.

Ultrasound-guided erector spinae plane block (ESPB) that aims for the ventral and dorsal rami of the spinal nerves was recently introduced in spine surgery to treat postoperative pain.^8^ ESPB was reported to be effective in scoliosis surgery as it reduced the need for analgesic drugs.^9–11^ Due to its relative technical simplicity, the main advantages postulated for the ESPB are few complications and minimal risk for spinal cord injury. However, only one trial concerning the influence of the ESPB on MEPs in scoliosis surgery was identified, and it applies to adult.^12^

Regional anesthesia, especially epidural anesthesia and/or analgesia, has been reported to inhibit the stress response and immune system dysfunction.^13^ The neuroendocrine system is activated during surgery and anesthesia, which results in the release of neuroendocrine hormones and cytokines. Postoperative pain during scoliosis surgery describes the control of neuropathic, nociceptive, and inflammatory pain and is associated with surgical stress response.^14^ Systemic leukocyte alterations, including neutrophilia leukocytosis and lymphopenia, occur in response to surgery.^15^ Nowadays, the neutrophil-to-lymphocyte (NLR) and platelet-to-lymphocyte (PLR) ratios are widely used as readily available and reliable markers of immune response to various noninfectious stimuli.^16^ NLR is not only affected by surgical trauma but also by the anesthetic method.^17,18^ However, very few studies have been conducted to evaluate the effects of different methods of anesthesia on NLR.^15,19,20^ Furthermore, until today, no trial has been shown to assess the impact of peripheral nerve block on NLR following pediatric surgery.

## AIM

This randomized, double-blinded, controlled trial was set out to assess the effect of ultrasound-guided ESPB on postoperative pain, morphine requirements, MEPs, NLR, and PLR to determine the difference in pain control and stress response versus standard analgesia in pediatric scoliosis surgery.

## METHODS

**FIGURE 1:**
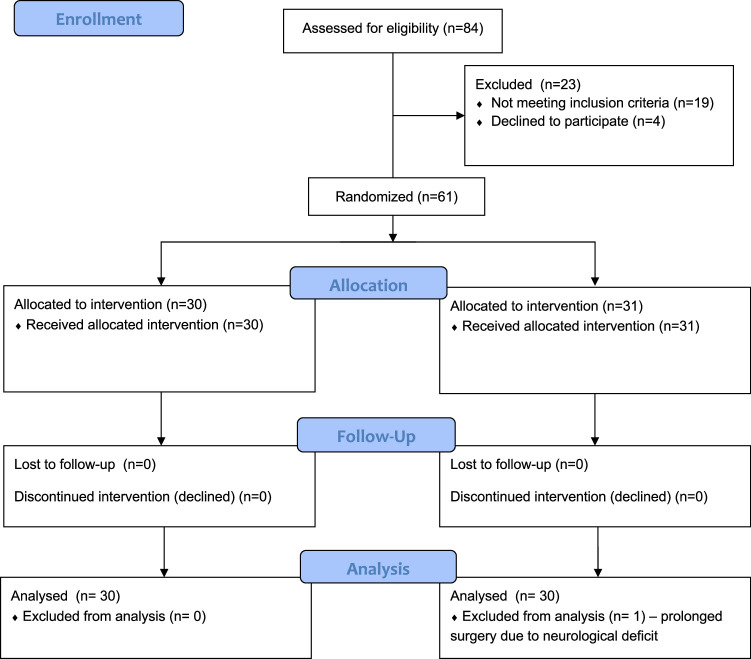
Flowchart of the study.

According to the Good Clinical Practice Guidelines (GCP) and the Declaration of Helsinki, this was a prospective, double-blinded, randomized controlled trial in 2 parallel groups. Our Institutional Reviewer Board reviewed and approved the trial (protocol number 673/22). We followed the Consolidated Reporting Trials Standards (CONSORT), as shown in Figure 1. Enrollment occurred from September 10, 2022 to May 12, 2023. The study was performed at the Orthopedic and Rehabilitation Hospital of Poznan University of Medical Sciences. Enrolment was offered preoperatively to patients undergoing posterior spinal instrumentation and fusion due to idiopathic scoliosis, aged more than 10 and less than 18 years, and ASA physical status 1 or 2. Parents/caregivers’ written informed consent was obtained in each case.

Patients were not included in this study: if they refused to participate, had a history of chronic pain (use of gabapentin/pregabalin for >3 mo or opioid use >1 repeated opioid prescription in the last 3 mo), had an infection of the puncture site, had abnormal coagulation test values, were aged less than 10, or more than 18 years, or were assessed as having ASA>2. We also excluded patients with a history of allergy or contraindication to the study drugs or ESPB.

### Randomization

Patients were randomly allocated to receive ultrasound-guided ESPB, or sham block, by computer software 1:1 (http://www.randomizer.org).^21^

The double blinding in this study was fulfilled with the precise designing of the work tasks for the researchers who were unaware of each other’s final scores. The first researcher not involved in the study prepared the randomization list and concealed group assignments consecutively numbered, sealed, opaque envelopes. Another consultant anesthesiologist followed management to open the envelopes shortly before the regional anesthesia performance to reveal the group allocation and perform the procedure according to the assignment. As a result, patients, surgeons, operating room staff, and the anesthesia team were masked to the study group allocation. Group blinding unmasking occurred once the statistical analysis was complete.

All patients underwent surgery consisting of posterior spinal correction using segmental instrumentation and fusion performed under general analgesia by one surgical team. All participants were instructed on how to self-report pain using the Numerical Rating Pain Score (NRS) (range, 10—maximum pain, 0—no pain) and how to operate a patient-controlled analgesia (PCA) device. The evaluation was repeated during the subject’s examination by 2 independent physicians encouraging the subjects to describe the pain intensity numerically at certain stages of observation. The final score was agreed upon at the end of the examination set.

### Procedures

In both groups, the patients received 7.5 mg of midazolam orally and 0.1 mg/kg of dexamethasone intravenously half an hour before the procedure, as a part of multimodal preemptive analgesia. General anesthesia with endotracheal intubation and volume-controlled ventilation (O_2_/air 40:60) was induced with 2.5 to 3.5 mg/kg of propofol and 2 μg/kg fentanyl intravenously. Then, endotracheal intubation was facilitated with 0.6 mg/kg of rocuronium. Anesthesia was maintained using intravenous infusions of propofol 4 to 12 mg/kg/h and remifentanil 0.5 to 1.5 μg/kg/min, titrated to achieve hemodynamic stability monitored through radial artery line, and adequate anesthetic depth monitored by Bispectral Index (BIS; GE Healthcare, Helsinki, Finland) values between 45 and 65. In addition, the combination of intraoperative 15 mg/kg acetaminophen, 15 mg/kg metamizole, and 10 mg/kg ibuprofen was applied as a multimodal analgesia protocol in the opioid-sparing anesthetic regimen.

**FIGURE 2:**
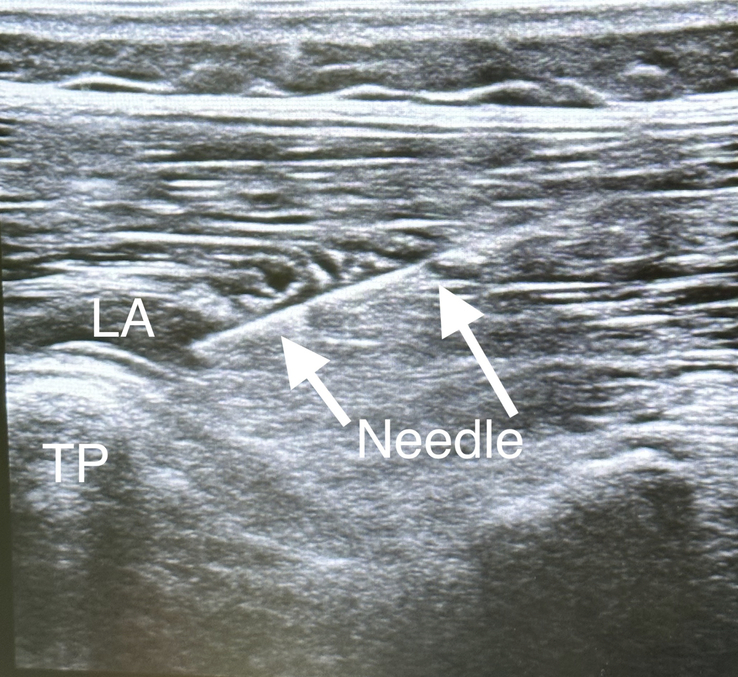
Erector spinae plane block (ESPB) technique.

After the induction of general anesthesia, bilateral, bilevel ESPBs were performed at Th4 and Th10 vertebral levels by an experienced regional anesthesiologist, as seen in Figure 2. In each ESPB, a 22 G needle (Stimuplex Ultra 360, 80 mm) was inserted into one plane of linear array ultrasound transducer [Mindrey TE9; Shangai International Holding Corp. GMBH (Europe), Hamburg, Germany] longitudinally positioned across the apex of the transverse process. The hand was directed caudally at the higher level and cranially at the lower level. Penetration of the fascial plane between the transverse process and the erector spinae was confirmed sonographically by hydro-location with 1 and 2 mL of 0.9% isotonic saline, followed by 10 mL injections of 0.2% ropivacaine using an in-plane technique.

#### During the Surgery

The basic hemodynamic parameters, opioid/propofol consumption, the MEPs’ amplitude, and the time of the surgery were monitored.

Postoperative analgesia consisted of intravenous acetaminophen 15 mg/kg 6-hourly, 15 mg/kg metamizole 6-hourly, and 10 mg/kg ibuprofen 6-hourly administered simultaneously to prevent rebound pain. In addition, a bolus of 0.1 mg/kg of morphine sulfate was administered for rescue analgesia if the Numerical Rating Scale (NRS) score was higher than 4, followed by a continuous infusion of 0.2 mg/kg/h of morphine sulfate as patient-controlled anesthesia (PCA).

At all postoperative time points (30, 60, 90, 120 min; and 6, 12, 24, and 48 h), patients were asked to rate perceived pain using a NRS 0 to 10 (0 indicating no pain and 10 indicating the worst pain imaginable) experienced during motion. Total opioid consumption and time to first opioid use were obtained from the postoperative unit and pediatric orthopaedic ward. Blood samples for NLR and PLR were collected 12 and 24 hours postoperatively.

The outcome assessment was performed by 2 clinicians who were blinded to the group allocation.

### Sample Size and Statistical Analysis

To calculate the sample size, we considered our primary hypothesis that the ESPB improves pain management. The sample size was calculated for the paired *t* test. Depending on previous trials with a 2-tailed type I error of 0.05, and power of 80%, and an effect size factor of 0.5 should involve 58 or more subjects.^22^ We evaluated 10% more patients in both groups to compensate for potential dropouts.

Statistical analysis was performed using GraphPad Prism 8 software (GraphPad Software Inc., San Diego, CA).

The parametric distribution of numerical variables was evaluated using the Kołmogorov-Smirnov normality test. The Student *t* or Mann-Whitney *U* tests assessed differences between groups. Categorical variables were compared with the Mann-Whitney *U* test, and an analysis of contingency was compared with the Fisher exact test. A P-value <0.05 was considered significant.

## RESULTS

Of 84 patients assessed for eligibility, 19 did not meet the inclusion criteria, and 4 declined to participate. The remaining 61 were randomly allocated between groups. One patient was excluded for prolonged surgery time due to neurological complications, as shown in Figure 2. The remaining 60 were analyzed. No clinically relevant differences were apparent from group characteristics, as shown in Table 1.

**TABLE 1:** Baseline Characteristics

	ESPB group (N=30)	Sham group (N=30)	*P*
ASA	1.5 (0.5)	1.6 (0.5)	0.2474
Age (y)	13.5 (1.81)	13.3 (1.57)	0.5142
Sex (F/M)	15/15	14/16	0.5627
Weight (kg)	47.0 (9.9)	52.4 (15.9)	0.1160
Height (cm)	156.1 (8.8)	156.0 (12.9)	0.9712
Cobb angle (deg.)	61 (8.9)	65 (11.7)	0.1499
Surgery duration (min)	249.0 (36.8)	252.7 (64.7)	0.8007
Operated vertebrae levels	10.5 (1.2)	10.5 (1.2)	0.9826

Values are mean (SD) or n/n.

ASA indicates American Society of Anesthesiology; ESPB, erector spinae plane block; F, female; M, male.

ESPB patients had lower NRS scores at all time points (30, 60, 90, 120 min; and 6, 12, 24, and 48 h after surgery), all *P*<0.0001, as seen in Table 2.

**TABLE 2:** Primary Study Outcomes

	ESPB (N=30)	Sham (N=30)	*P*
NRS postoperative			
30 min	1.3 (1.0)	4.2 (1.1)	<0.0001
60 min	1.4 (1.0)	4.5 (1.1)	<0.0001
90 min	2.0 (1.4)	4.0 (1.3)	<0.0001
120 min	2.0 (1.5)	3.9 (1.0)	<0.0001
6 h	2.1 (1.6)	4.0 (0.9)	<0.0001
12 h	1.8 (1.3)	3.6 (1.0)	<0.0001
24 h	1.6 (1.2)	3.6 (0.8)	<0.0001
48 h	1.5 (0.9)	3.1 (0.8)	<0.0001

Values are mean (SD) or numbers.

ESPB indicates erector spinae plane block; NRS, Numerical Rating Scale.

The need for remifentanil (μg/kg/h) during surgery was lower in the ESPB group (123.77±8.5 vs. 261.3±29.3; *P*<0.0001). Likewise, the need for propofol (mg/kg/h) was lower in the ESPB group (0.50±0.1 vs. 1.1±0.2; *P*<0.0001). Therefore, total opioid consumption (intravenous morphine equivalents in mg/kg) was lower at all time points, all *P*<0.0001, as seen in Table 3. Also, the incidence of nausea or vomiting was lower in the ESPB group (0% vs. 47%; *P*<0.0001).

**TABLE 3:** Secondary Study Outcomes

	ESPB (N=30)	Sham (N=30)	*P*
Drugs during surgery			
Propofol (mg/kg/h)	0.5 (0.1)	1.1 (0.2)	<0.0001
Remifentanil (μg/kg/h)	124.8 (8.5)	261.3 (29.3)	<0.0001
Total opioid consumption (intravenous morphine equivalents; mg/kg)			
0-24 h	0.3 (0.1)	1.9 (0.3)	<0.0001
24-48 h	0.3 (0.1)	1.6 (0.3)	<0.0001
48-72 h	0.2 (0.1)	1.3 (0.3)	<0.0001
72-96 h	0.1 (0.1)	1.0 (0.3)	<0.0001
96-120 h	0.1 (0.1)	0.7 (0.3)	<0.0001
120-142 h	0.0 (0.0)	0.4 (0.2)	<0.0001
142-164 h	0	0.1 (0.1)	<0.0001
Nausea and vomiting			
Yes (%)	0	47	<0.0001
NLR			
Before surgery	1.7 (1.6)	1.5 (0.8)	0.9138
12 h	16.7 (10.3)	31.2 (12.8)	<0.0001
24 h	3.4 (1.3)	5.4 (4.2)	0.0072
PLR			
Before surgery	140.8 (58.7)	137.6 (40.6)	0.8456
12 h	349.6 (112.6)	541.0 (147.5)	<0.0001
24 h	129.3 (60.7)	234.5 (77.6)	<0.0001

Values are mean (SD) or numbers.

ESPB indicates erector spinae plane block; NLR, neutrophil-to-lymphocyte ratio; PLR, platelet-to-lymphocyte ratio.

The NLR was significantly lower in the ESPB group at 12 hours (16.66±10.30 vs. 31.20±12.84; *P*<0.0001) and at 24 hours (3.36±1.28 vs. 5.36±4.22; *P*=0.0072) after surgery. Also, PLR was lower in the ESPB group at 12 hours (349.57±112.63 vs. 540.96±147.54; *P*<0.0001) and at 24 hours (129.29±60.71 vs. 234.53±77.55; *P*<0.0001) after surgery, as seen in Table 3.

Before surgical scoliosis correction, the MEPs’ amplitude was 1311.7±92.3 γV in the ESPB group and 1329.5±125.3 γV in the sham group (*P*=0.06). After surgical correction of scoliosis, the MEPs’ amplitude was 1625.3±78.5 γV in the sham group and 1825.9±92.4 γV in the ESPB group (*P*=0.04), as seen in Table 4. The BIS value was similar in both groups (78.5±12.5 vs. 51.9±10.2; *P*=0.07), as seen in Table 4.

**TABLE 4:** Data of the Motor-Evoked Potentials (MEPs) Amplitude

	Mean±SD		
	ESPB group (N=30)	Sham group (N=30)	*P*
MEP amplitude (γV) before surgical scoliosis correction	1311.7±92.3	1329.5±125.3	0.06
MEP amplitude (γV) after surgical scoliosis correction	1825.9±92.4	1625.3±78.5	0.04
Bispectral Index (BIS)	51.9±10.2	51.7±12.5	0.07

## DISCUSSION

In this double-blinded, randomized controlled trial (RCT) compared with sham block in thoracic scoliosis posterior surgery, the ESPB reduced pain ratings and total opioid consumption, lowered the incidence of nausea and vomiting, reduced the need for propofol during surgery, provided less surgical stress response expressed by the NLR and PLR, while did not impact MEPs amplitude.

ESPB is reported to block the dorsal and dorsal branches of the spinal nerves and provides adequate pain management in spine surgery. The ESPB reveals a wide range of cranial and caudal spread through the paraspinal muscles via a single injection, facilitating ESPB coverage at multiple vertebral levels.^23,24^ Therefore, based on the previous study,^25^ we chose the Th4 and Th10 levels as the ESPB injection site. In addition, in the ESPB local anesthetic is injected in the deep plane to the erector spinae muscles and superficial to the transverse processes to achieve a craniocaudal distribution along several vertebral levels.^8,26^ The erector spinae muscle includes the spinalis muscles, longissimus, and iliocostalis.^27^ These muscles pace bilaterally from the transverse processes, lengthening to the ribs and from the skull to the pelvis and sacral region.^26^ Sensory innervation to the upper posterior thorax derives from the first cervical over the fifth lumbar nerve. The ventral rami of the thoracic spinal nerves from the T1 to T12 continue as intercostal nerves innervating the anterolateral chest and abdominal wall.^28,29^ Mechanisms of nerve blockade and distribution of the local anesthetic are not coherent. Studies suggest that LA, after an ESPB, disperses in a cephalocaudal distribution and attains dorsal, ventral, and dorsal rami of the spinal nerves and intercostal spaces.^30–33^

On the other hand, epidural analgesia with local anesthetics and/or opioids is frequently performed after scoliosis surgery. Continuous analgesia through 1 or 2 epidural catheters placed by the surgeon at the end of the procedure has provided efficient postoperative pain control after scoliosis correction.^34,35^ However, high failure rates and complications after epidural catheters like hematoma and epidural or vascular puncture are rare but have profound implications.^36,37^

Ultrasound-guided peripheral nerve blocks reduce these risks and thus should be favored, especially in large spine surgeries.

As far as we know, this study is the first prospective, randomized trial on the ESPB application for pediatric scoliosis surgery. Hitherto, Akesen et al,^25^ in their retrospective study, revealed that VAS scores and postoperative cumulative morphine consumption within the first 24 hours were significantly lower in adult patients with ESPB. Also, ESPB provided adequate postoperative analgesia in adult patients undergoing lumbar spine surgery.^38^ Our results are similar to these 2 studies. Therefore, it can be concluded that preoperative ESPB was revealed to be effective in postoperative pain management after pediatric thoracic scoliosis posterior surgery.

All ESPB patients in our study did not experience postoperative nausea or vomiting, probably due to lower opioid consumption. We have not identified any similar study in children. However, in the adults, the incidence of nausea and vomiting was reported to be lower with ESPB.^8^

These results present relevant clinical implications. The ESPB-related sufficient pain control and lower need for opioids promote early mobilization and rehabilitation and prevent the development of persistent postoperative pain.

Long-term general anesthesia is needed for posterior scoliosis correction and fusion. Total intravenous anesthesia (TIVA) using remifentanil and propofol is recommended for spine surgery.^39^ However, propofol infusion might be associated with developing propofol-related infusion syndrome (PRIS), a lethal condition characterized by multiple organ system failures. PRIS can create several serious adverse effects, including myocardial failure, cardiac asystole, metabolic acidosis, rhabdomyolysis, and death.^40^ Heavy or extended dosage of propofol is one of the main risk factors predisposing to PRIS. The PRIS mainly occurs in intensive care units but was also observed in children after general anesthesia.^41,42^ As demonstrated by this study, ESPB allows for reducing propofol and remifentanil infusion during spine surgery, which is particularly important in preventing the development of PRIS, in children with neuromuscular disease.^43^

Neurophysiological monitoring of spinal cord integrity by MEP (motor-evoked potential) and SSEP (somatosensory-evoked potential) are used for scoliosis surgery safety.^44,45^ The anesthetic drugs, including remifentanil and propofol, inhibit the amplitudes of transcranial motor-evoked potentials in a dose-dependent manner.^46^ Therefore, the neurological safety of scoliosis surgery requires minimalizing the infusion of anesthetic drugs, as reported in our study with ESPB. However, Chin and El-Boghdadly^47^ claimed that ESPB local anesthetics might spread to the epidural and/or paravertebral space, which would be a potential concern for ESPB interference with MEPs during spine surgery. However, the confirmation of this hypothesis is not studied in detail. In a case report, Stondell and Roberto^10^ reported a unilateral transient substantial MEP decrease. We did not observe any ESPB-related MEPs disturbances. Our preliminary observations suggest a relationship between the intensity of stimulus strength for evoking MEPs and the level of bispectral index monitoring (BIS): optimal MEPs recordings once BIS was kept at the 55 range.

It was reported that continuous anesthesia during a 4-hour lasting spine surgery influences the amplitude more than the latency parameter of the intraoperatively MEPs. In our experience, adding ESPB to standard anesthesia protocol decreased the required anesthetic volume and did not influence the MEP’s recording.

Surgical site infection (SSI) is one of the most severe complications of spine surgery. SSI can result in reoperation and extended hospital stay and is associated with considerable morbidity. Neutrophil-to-lymphocyte ratio (NLR) is widely used as a marker of immune response to infectious and noninfectious stimuli. NLR reflects the dynamic relationship between innate (neutrophils) and adaptive (lymphocytes) cellular response to surgery.^48^ NLR correlates with SSI^49^ and identifies patients at higher risk of SSI after spinal surgery in adults.^50^ After surgery for spinal degeneration, adult patients with higher NLR and platelet-to-lymphocyte ratio (PLR) values have developed postoperative deep vein thrombosis.^49^ From the age of 3 to 18 years, the 50th percentile of NLR is constantly increasing from 0.99 to 1.76 for both females and males, and the NLR values over 3.0 are considered pathologic.^51^ Also, the PLR values constantly increase from 60 at 3 years to 120 at 18 years.

Regional anesthesia, especially spinal anesthesia, lowers the NLR levels in adults^15,19,52^; however, no study considered the pediatric population. Tantri and colleagues^1–3,14^ showed that ESBP decreases the interleukin-6 and interleukin-10 concentrations, thus lowering the stress response during lumbar surgery in adults. There is no similar study on children. Our analysis indicates ESPB reduces the surgical stress response expressed through NLR and PLR. Therefore, ESPB lowers the risk of SSI and deep vein thrombosis (DVT) following scoliosis surgery.

Our study is the first to describe the effects of peripheral nerve block on NLR and investigate the impact of peripheral nerve block on surgical stress response in children.

The main limitation of this study is the sample size. Also, the dermatome levels were not evaluated. Another limitation is that we did not obtain NLR and PLR over 24 hours. Finally, we did not monitor the hospital discharge time.

## CONCLUSIONS

Based on our findings, ultrasound-guided bilateral and bilevel ESPB at the thoracic level lowers the demand for remifentanil and propofol during scoliosis surgery, reducing the anesthesia influence on intraoperative MEPs amplitude. ESPB lowers the NLR and PLR, thus reducing the stress response. Also, ESPB is effective for postoperative analgesia and can reduce opioid consumption in patients undergoing scoliosis surgery. Therefore, we recommend this technique as a part of multimodal analgesia protocols in adolescent scoliosis surgery.
